# Engineering-geological comparative analysis of four cases studies of waste landfills

**DOI:** 10.1038/s41598-023-36790-1

**Published:** 2023-06-21

**Authors:** Marian Marschalko, Dariusz Popielarczyk, Petr Vicherek, Dominik Niemiec, Jan Kubac

**Affiliations:** 1 Department of Geological Engineering, Faculty of Mining and Geology, VSB-Technical University of Ostrava, 17 listopadu 15, 708 33 Ostrava, Czech Republic; 2 Department of Geodesy, Faculty of Geoengineering, University of Warmia and Mazury in Olsztyn, Oczapowskiego 2, 10-719 Olsztyn, Poland

**Keywords:** Ecology, Environmental sciences, Natural hazards, Solid Earth sciences

## Abstract

The aim of the paper is to carry out a comparative engineering-geological study of four different waste landfills using the evaluation criteria for the geological subsoil as a natural sealing barrier. The study evaluates 4 localities (Velké Pavlovice, Kvítkovice, Prakšice and Horní Suchá) using three variants (based on two standards) which approach the geological barrier requirements as a combination of impermeability requirements based on a filtration coefficient limit value. and the required geometry represented by investigation depths. The research was carried out in landfills in Moravia, in the east of the Czech Republic. The study’s motivation is to point at the differences in engineering-geological investigations of waste landfills (as for the requirements for impermeable geological subsoil as a natural sealing barrier) when compared with other engineering structures (where the main goal is to evaluate load-bearing capacity and settlement). The purpose of the geological barrier is to prevent the spread of contamination, and the paper shows this can be approached differently, as shown in two different methodologies investigated herein. The first model (Model 1) assumes there is a 3-m-thick subsoil below the landfill’s footing bottom, which manifests impermeability characterized by the filtration coefficient K_f_ ≤ 1.0 * 10^–9^ m/s, or a 30-m-thick subsoil of K_f_ ≤ 1.0 * 10^–8^ m/s. The second model (Model 2) assumes a 1-m thick, impermeable subsoil massif of K_f_ ≤ 1.0 * 10^–9^ m/s. We found that none of the landfills in the four selected localities had an impermeable layer in the required depth (a filtration coefficient K_f_ from 1.8 * 10^–9^ to 3.9 * 10^–9^ m/s), and thus did not comply with the limiting conditions. As a result, an anthropogenic technical barrier had to be installed. An important goal of the study from an environmental point of view was to assess the existence of a suitable geological barrier under the proposed landfills. The most important criterion from this point of view is permeability. An additional technical objective of the project was also the assessment of the possible creation of a technical anthropogenic isolation barrier. In the event that the natural sealing barrier would not be sufficient. This was shown in all solved case studies of engineering geological investigations of waste landfills.

## Introduction

The paper deals with engineering-geology investigations related to *waste landfills*, which is a specific engineering geology problem in the sense of investigating the *required geological barrier*^1–4^ and related requirements for *impermeability and sufficient layer thickness*. While engineering geology studies are mostly concerned with settlement and load-bearing capacity solutions^5–10^ in engineering structures, the fundamental problem in waste landfills is to prevent potential contamination from landfills^11–14^ to spread into the subsoil, to avoid ground water contamination^15–19^, or to avoid the spread of contamination in general. Not only the choice of the site for a waste landfill is crucial, which is discussed in^20–24^, but engineering-geological investigations of waste landfill sites play a big role^2,25–30^ in connection with aptly applied methods and geophysical surveying^31–33^.

The problem of waste landfills is mainly related to preventing *potential contamination* by the materials disposed of in the landfill, i.e., a problem that needs to be eliminated. The *first solution* lies in a geological subsoil in a certain geometry below the landfill, which complies with the requirements for sufficient impermeability. The *second solution* lies in using an anthropogenic sealing barrier. The *third solution* combines the first and second one.

The ability of a porous geological environment to transmit liquids with certain properties (e.g. groundwater) is called permeability. If we relate permeability to flow, we can express its rate by means of the *filtration coefficient*. The filtration coefficient expresses the degree of permeability of the geological environment. It is the ratio of the water flow rate (filtration rate) to the hydraulic gradient. The filtration coefficient was determined for individual soil samples from the footing bottom waste dump by calculations based on grain size curves in an accredited laboratory using the Carman-Kozeny method. The filtration coefficients are necessary to properly plan and design the landfill sealing barrier for the potential existence of a so-called impermeable geological barrier. If the criteria are not met, the barrier cannot be counted on. If the geological barrier is identified and verified, the landfill sealing barrier is simpler and therefore less expensive.

*Impermeability* is determined based on the filtration coefficient parameters which correspond to a certain degree of permeability. Filtration coefficient has been discussed by a number of studies^34–38^, while impermeability and landfills remain understudied^39–41^. Impermeability was rather studied in connection with other engineering structures^42–45^ and the closest research question to waste landfills may be impermeability related to dam construction^46–48^.

*The issue of geometry* is approached having selected the depth of engineering-geological investigations aiming to evaluate the exploratory work results. The issue of depth in engineering-geological investigations was studied by^2^, Vest Christiansen and Auken (2012), but mainly in connection with another character of engineering-geological investigations.

To demonstrate engineering-geological investigations of waste landfill sites, we carried out a comparative study of 4 selected waste landfill localities (4 case studies) in Moravia, in the east of the Czech Republic. The research aims to contribute to better specification, characterisation and presentation of the phenomenon. The research results may reveal a subsoil that is not sufficiently thick and impermeable, and thus an anthropogenic barrier needs to be installed. When evaluating the issue of geological environment as a natural barrier for waste landfills, we may produce a number of concepts/models. This study will evaluate two models, where each is based on a different standard: Model 1- Standard CSN 83 8030^49^, Model 2—Standard CSN 83 8030^50^.

The study had environmental and technical objectives. In terms of environmental objectives, the most important was the assessment of the tightness of the geological barrier of the subsoil against possible contamination under the landfill object. This evaluation was carried out through an engineering geological investigation of four waste landfills, where the main evaluation criterion was the permeability of this natural sealing barrier. If we define the technical goals of the study, it is crucial to assess the possibility of creating a technical anthropogenic isolation barrier. This is in case the results of the engineering geological investigations show that the natural sealing barrier is not sufficient.

## Theoretical background of the study

**Figure 1:**
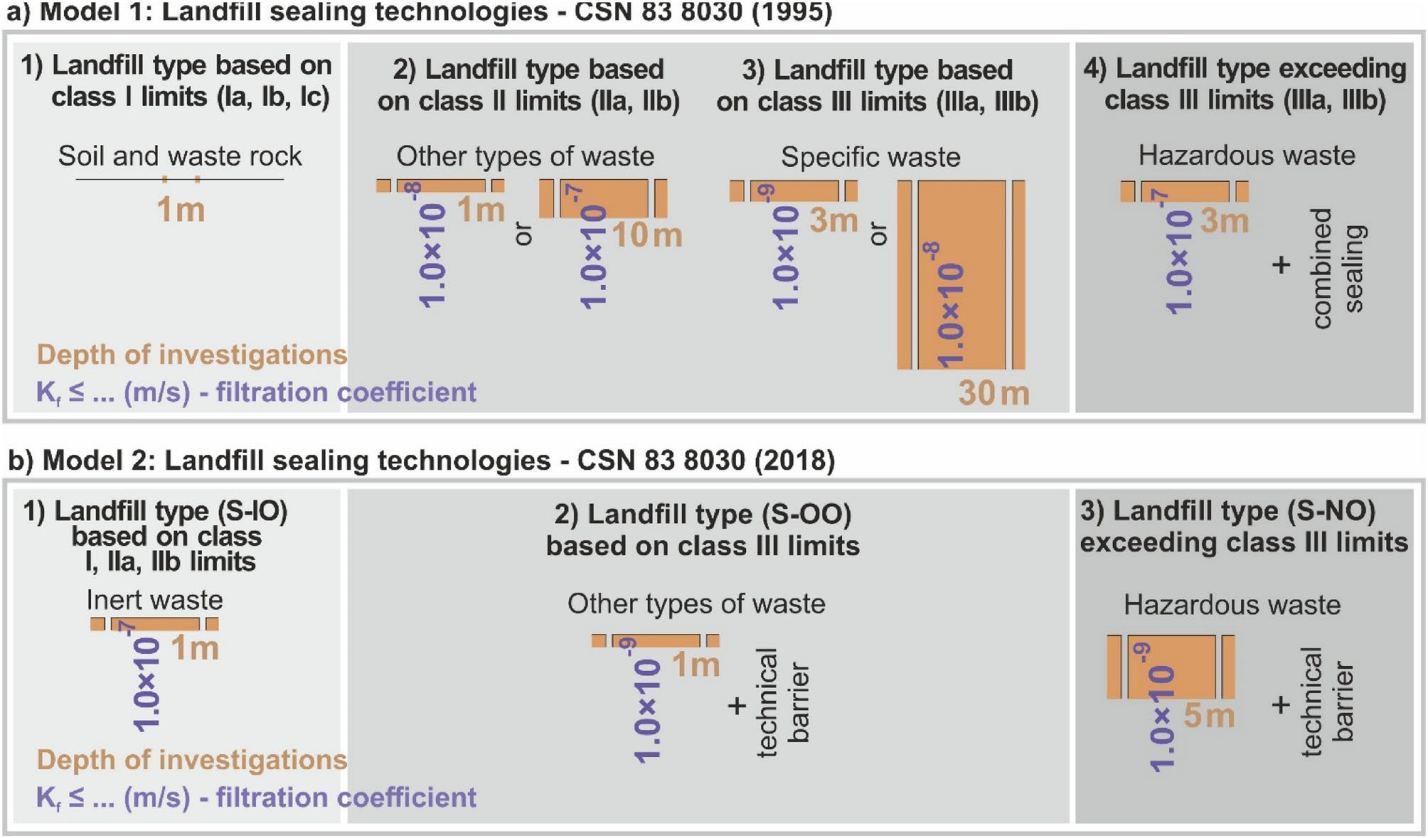
Requirements for the landfill’s geological subsoil (two parameters—the depth of the engineering-geological investigation, and the impermeability of the geological environment based on filtration coefficient) in the sense of the geological barrier for the disposal of waste based on landfill categories and the limit leachability values (Table 1); (**a)** Model 1 according to CSN 83 8030^49^ Standard, (**b)** Model 2 according to CSN 83 8030^50^ Standard.

Analyzing the issue of geological environment as a natural barrier to waste landfills we compared two models, where the Model 1 follows CSN 83 8030^49^ Standard and Model 2 follows CSN 83 8030^50^ Standard. Each of them shows one of the approaches to evaluate the suitability or unsuitability (or subsequent measures such as landfill barrier) of the geological environment from the point of view of the possibility of locating and establishing a landfill. Requirements for the landfill’s geological subsoil in the sense of the geological barrier for the disposal of waste based on landfill categories and the limit leachability values for both models presents (Fig. 1).

Waste leachability classes are a way of categorizing waste according to their ability to release hazardous substances into water. These classes are used to assess the risks associated with waste management, such as disposal in landfills. The leachability classes are divided starting from the least leachable materials to the most leachable materials. Evaluating the leachability of waste is therefore an important factor in determining the most appropriate waste management method to minimize environmental risks. This means that these leachability classes are used to determine the type of landfill to which the collected category of waste will be transported. The relationship between the various leachability classes (Table 1) and the requirements for the geological substratum of landfills can be seen in Fig. 1. Table 1 shows selected chemical criteria defined by the leachability classes for the subcategories in Model 1 and Model 2.

**Table 1:** Selected chemical criteria specified by leachability classes for subcategories in both the models.

Indicator	Model 1							Model 2			
	Leachability class							Leachability class			
	Ia	Ib	Ic	IIa	IIb	IIIa	IIIb	I	IIa	IIb	III
	mg/l	mg/l	mg/l	mg/l	mg/l	mg/l	mg/l	mg/l	mg/l	mg/l	mg/l
DOC (dissolved organic carbon)								50	80	80	100
Phenols								0.1			
Chlorides	200		100					80	1500	1500	2 500
Fluorides	1.5	3	1.5	5	5	20	50	1	30	15	50
Sulphates	250		250	0.1	1			100	3000	2 000	5 000
As	0.05	0.1	0.05	0.1	0.1	1	5	0.05	2.5	0.2	2.5
Ba	0.5	1	1	1	10	5	50	2	30	10	30
Cd	0.005	0.005	0.005	0.05	0.05	0.5	0.5	0.004	0.5	0.1	0.5
Cr total	0.05	0.1	0.05	1	1	10	50	0.05	7	1	7
Cu	0.1	1	0.1	1	1	10	10	0.2	10	5	10
Hg	0.001	0.002	0.001	0.005	0.005	0.05	0.05	0.001	0.2	0.02	0.2
Ni	0.1	0.1	0.1	0.5	0.5	10	50	0.04	4	1	4
Pb	0.05	0.1	0.05	0.5	0.5	2	10	0.05	5	1	5
Sb	0.05	0.1	0.01	0.1	0.1	1	5	0.006	0.5	0.07	0.5
Se	0.01	0.05	0.01	0.05	0.1	0.5	5	0.01	0.7	0.05	0.7
Zn	3	3	3	3	3	10	100	0.4	20	5	20
Mo								0.05	3	1	3
RL						10 000	20 000	400	8 000	6 000	10 000
pH	5.5–10	5.5–11	6.5–8	5.5–12	5.5–13	5.5–13	5.5–13		≥ 6	≥ 6	

**Figure 2:**
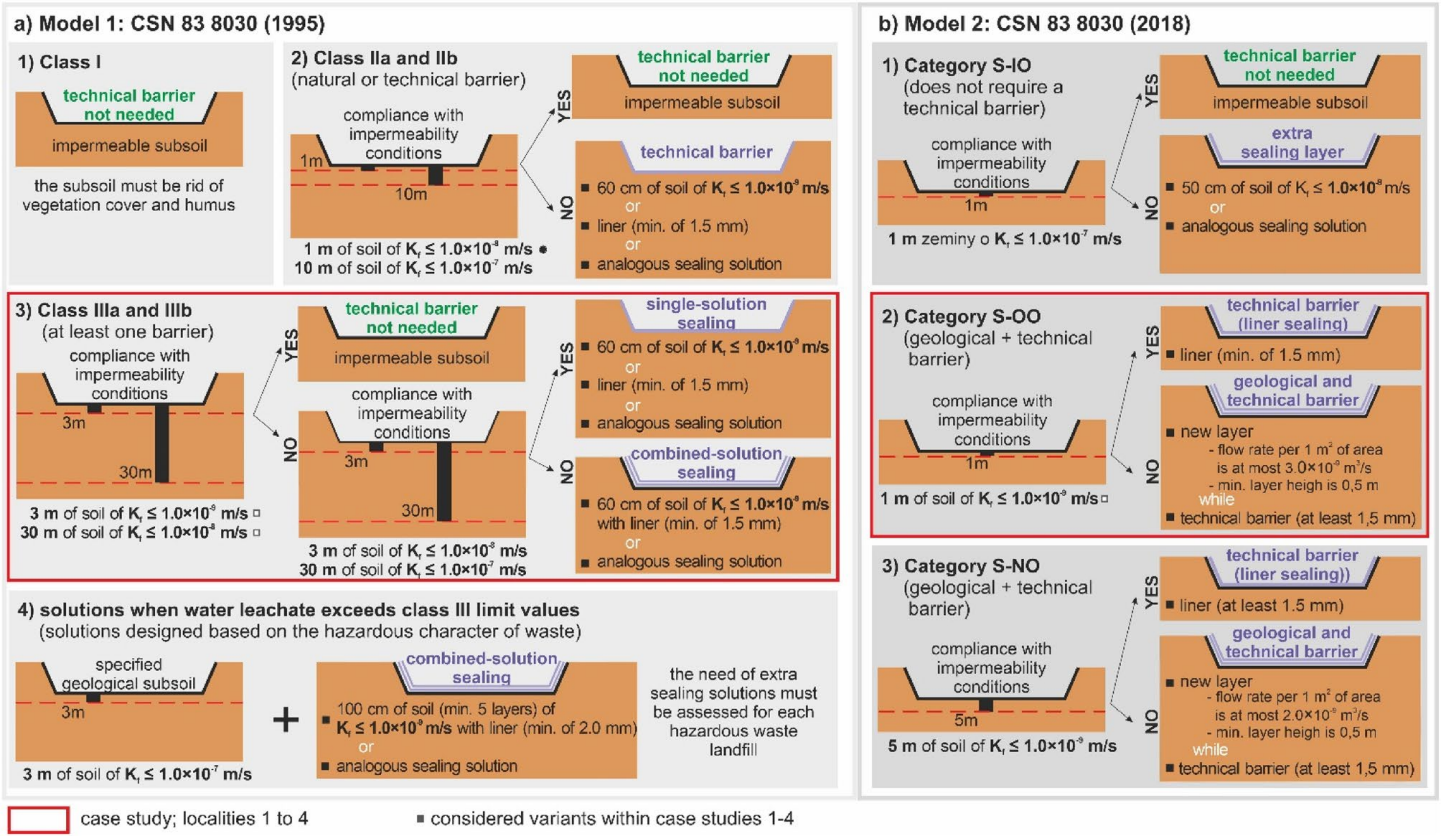
Conditions for applying technical barriers: (**a**) Model 1 according to CSN 83 8030^49^, (**b**) Model 2 according to CSN 83 8030^50^.

In terms of the impact of landfills on the surrounding environment, natural geological or anthropogenic barriers are a particularly important element of nature conservation. The purpose of a geological barrier is to stop the spreading of pollutants from the landfill. If the ground is not thick enough as a natural barrier, an anthropogenic barrier should be used. Conditions for applying technical barriers are presented in Fig. 2.

### Model 1

Model 1^51^ divides the engineering geological investigations into *4 categories* (Fig. 1a). *Category 1* is not important for this study as it does not address the relationship of subsoil impermeability and depth of investigation (there is no need for a natural geological barrier). In this category, subsoil impermeability is not required as it is the case of safe category waste (*soil and waste rock—“non-hazardous waste’*) and there is no contamination hazard. The category is characterized by leachability classes, while the waste leachability limits are strict (Table 1). As for the chemism (expressed by leachability class I), the category has 3 subcategories (Ia, Ib and Ic—Table 1). In the subcategory Ia, ground water must comply with the limit values set in leachability class Ia. When increased values of contaminated substances content are detected in ground water in the waste disposal site locality, limit values are decided according to subcategory Ib. When waste gets into contact with ground water, the waste must meet the limit values in subcategory Ic.

*The waste landfill category 2* already includes requirements for a geological barrier because there may be a risk of contamination related to the disposed waste. However, the risk is much lower than in the higher categories (categories 3 and 4). Category 2 includes waste landfills with chemism requirements for water leachability class II. In contrast to the first subcategory’s limits (see subcategory IIa in Table 1), in subcategory IIb increased values of contaminated substances content may be detected in the ground water (due to ground natural water genesis in the geological environment) and thus the subcategory allows for certain increases in the chemism values (see subcategory II b in Table 1).

In category 2, the requirement for the geological barrier has two variants (these variants are not related to subcategories IIa and IIb (Table 1) and thus apply to both subcategories. The *first variant* represents a situation when engineering-geological investigation checks the geological structure as deep as 1 m, while the geological environment’s impermeability, as expressed by filtration coefficient, will be lower than or equal to 1.0 * 10^–8^ m/s. The *second variant* represents a situation when engineering-geological investigation examines the geological structure at least 10 m deep and the geological environment’s impermeability as expressed by the filtration coefficient is lower than or equal to 1.0 * 10^–7^ m/s. The second variant is stricter with regard to the depth of the investigation as the impermeability requirement is lower (K_f_ ≤ 1.0 * 10^–7^ m/s as opposed to K_f_ ≤ 1.0 * 10^–8^ m/s).

*Waste landfill category 3* is a more hazardous category and the principle explained above (for category 2) applies also to this category. This means that category 3 has two subcategories IIIa, and IIIb (Table 1). Subcategory IIIb includes higher limit values when ground water in the locality does not comply with the limit values in IIIa, but there is a condition that limits may be exceeded (contrary to category 2) only in three chemisms.

If we evaluate the criteria that the landfill’s geological subsoil must comply with in the sense of a geological barrier, there is an analogy with Category 2. The difference is in the depth of the engineering-geological investigations, which is the minimum of 3 m (it is 1 m in Category 2), while the minimal impermeability expressed as a filtration coefficient is lower than or equal to 1.0 * 10^–9^ m/s. Alternatively, investigations must be as deep as 30 m (contrary to 10 m in Category 2), and the minimal impermeability expressed as a filtration coefficient must be lower than or equal to 1.0 * 10^–8^ m/s.

Waste in the *waste landfill category 4* is the most hazardous—see Table 1, and thus there are much stricter requirements for the so-called combined anthropogenic sealing of landfills. The requirements concern the landfill structure and the insulation capacities of the geological subsoil. Because of the technical sealing solution (the landfill is highly insulated), the requirements for the subsoil are less strict than in the previous variants.

As for the geological subsoil, the investigations are carried out as deep as 3 m. The subsoil impermeability requirements state that the filtration coefficient must be lower than or equal to 1.0 * 10^–7^ m/s. At the same time, the subsoil requirements cannot be compared with the two previous variants because it is the case of a combined solution of a natural geological and technical (anthropogenic) landfill barrier.

### Model 2

When evaluating the Model 2 (Fig. 1b) of geological barrier requirements for landfills in line with CSN 83 8030^50^ Standard, we can distinguish 3 categories of requirements. The analogy between the Model 1 and the Model 2 is that Category 1 in the first model is the least hazardous waste, and the most hazardous waste is associated with the last category of each model.

#### Waste category 1

Figure 1b1 of the Model 2 is characterized by inert waste (more or less corresponds to Category 1 in the first model). It represents the most favorable variant in terms of waste hazardous properties (inert waste is the least hazardous) and the requirements for the geological barrier quality. The required depth of the geological investigations is low (1 m) and the requirement for impermeability is the lowest (K_f_ ≤ 1.0 * 10^–7^ m/s) out of all the 3 categories.

#### Waste category 2

Figure 1b2 concerns somewhat hazardous waste (other waste, more or less corresponds to Categories 2 and 3 of the Model 1, and subcategories determined based on leachability classes IIa, IIb according to Table 1). Although the depth of the geological investigations remains unchanged (1 m as in Category 1), the difference is in the stricter requirement for the impermeability of the geological environment, i.e., K_f_ ≤ 1.0 * 10^–9^ m/s. In addition, contrary to the Model 1, there is also a requirement for a technical barrier, which calls for the so-called combined solution of insulation barriers (see Introduction Section). This way, the requirement for more shallow investigations is compensated for by the use of a landfill barrier.

#### Waste category 3

Figure 1b3 is the most hazardous group of wastes, which manifests in the need for the deepest engineering-geological investigations (5 m) as opposed to 1 m in the previous two categories. At the same time, there are higher demands for impermeability. Although the filtration coefficient is identical with Category 2 (K_f_ ≤ 1.0 * 10^–9^ m/s), there are stricter requirements for the depth of the impermeable subsoil (5 m). Moreover, the requirements for the geological barrier are complemented by a technical barrier. The specification of the technical barrier is explained in Fig. 2. If we compare this category with the analogous category of the first model (Category 4), the difference is only in the increased requirements for the landfill barrier.

In *Waste landfill category 1* (Model 1) there is no need for a technical barrier because of the inert character of the waste (soil and waste rock) (Fig. 2a1).

If the criteria for the geological barrier are met in *Waste landfill category 2* (Fig. 2a2), there is no need to install a technical barrier. If not, a technical barrier of 60-cm soil layer of K_f_ ≤ 1.0 * 10^–9^ m/s must be incorporated. Alternatively, the second option is to install a liner (minimum thickness of 1.5 mm) in the footing bottom of the landfill. A different solution is possible if the requirements of both technical barriers are met.

There is no need for a technical barrier in *Waste landfill category 3* if the criteria are met (Fig. 2a3). However, if these are not met, the technical barrier must be installed. In contrast to Waste landfill category 2, in Waste landfill category 3 the geological barrier must partially meet the impermeability criterion. There are analogous depths as above, but the impermeability parameters (filtration coefficient) are stricter. The subsoil as deep as 3 m must comply with K_f_ ≤ 1.0 * 10^–8^ or the subsoil as deep as 30 m must comply with K_f_ ≤ 1.0 * 10^–7^.

If the requirements for the geological barrier are met, the technical barrier solutions are analogous to waste landfill category 2. This means that a 60-cm-thick layer of subsoil (K_f_ ≤ 1.0 * 10^–9^ m/s) is required, or a liner (minimum thickness of 1.5 mm) must be installed (or a similar barrier with analogous sealing effect). If the stricter requirements for the geological barrier are not met, there is a need for a technical barrier with combined insulation. This means both the above-mentioned solutions are combined (60-cm soil layer of K_f_ ≤ 1.0 * 10^–9^ m/s and a 1.5-mm-thick liner), or a similar barrier of analogous sealing effect is installed (Fig. 2a3).

*Waste landfill category 4* (Fig. 2a4) represents a category where the water leachate class III limits are exceeded. In this category we need to use a different strategy of landfill insulation. The role of the technical barrier (minimum thickness of 2 mm) is crucial here with the combined solution of 100-cm layer of impermeable soil of K_f_ ≤ 1.0 * 10^–9^ m/s. Not only the sealing subsoil layer is thicker, also the liner is thicker.

As for Model 2, *Category 1* (inert waste landfill, Fig. 2b1) has the following criteria for the geological barrier: 1-m layer of soil of K_f_ ≤ 1.0 * 10^–7^ m/s. If these are met, there is no need for the technical barrier. If not, the technical barrier must consist of a 50-cm-thick layer of soil of K_f_ ≤ 1.0 * 10^–8^ m/s., or there is need to be a barrier of analogous sealing effect.

*Category 2* (landfill for other types of waste, Fig. 2b2) must consist of two barriers (geological and technical). If the criteria are met for the first barrier (soil massif of K_f_ ≤ 1.0 * 10^–9^ m/s as deep as 1 m), the technical barrier requirement is a liner (minimum thickness of 1.5 mm). If not, the geological barrier must be complemented by an extra 0.5-m layer of soil (K_f_ ≤ 3.0 * 10^–9^ m^3^/s). In well-justified cases analogous sealing solutions may be used. If this extra layer (0.5 m) does not meet the minimum thickness requirement, a monitoring system must be installed (in line with Standard CSN 83 8036) to monitor the compactness of the geological and technical barriers. Monitoring is carried out as long as the layer of waste disposed in the landfill reaches 2 m. The geological barrier (including the extra layer) must be complemented by a liner (minimum thickness of 1.5 mm).

In *Category 3* (hazardous waste landfill, Fig. 2b3) the solution comprises at least two barriers as in Category 2. There are analogous requirements for impermeability (K_f_ ≤ 1.0 * 10^–9^ m/s) as in Category 2, but the difference is in the depth of the engineering-geological investigations is bigger (5 m as opposed to 1 m in Categories 1 and 2). If the criteria for the geological barrier are met, the technical barrier is the same as in Category 2, i.e., a liner of 1.5 mm. If not, there is an analogy with the solution in Category 2. This means we need to install an extra layer (thickness of 0.5 m) which must have filtration coefficient K_f_ ≤ 2.0 * 10^–9^ m^3^/s (it was 3.0 * 10^–9^ m^3^/s in Category 2). Besides the identical requirement for landfill monitoring as in category 2, there is an extra requirement to assess the landfill for the need of potential protective barriers or sealing features.

If we compare Model 1 and Model 2 from the perspective of the solved case studies (Fig. 2a3—Model 1 and Fig. 2b2—Model 2), there are two fundamental differences. The first difference is that the current standard (CSN 83 8030^50^—Model 2) is not as strict with regard tp the strength of the impermeable geological subsoil (natural geological barrier) as the older standard (CSN 83 8030^49^—Model 1). The second significant difference is that the current standard (Model 2) always recommends the construction of a technical barrier. Whereas Model 1 (the older standard) does not always have to build a technical barrier, but only if the natural geological barrier does not meet certain requirements.

## Study area

An engineering-geological comparative analyses was carried out on landfills using the criteria of evaluating the geological subsoil as a natural sealing barrier. The study analysed four landfills in Moravia, in the east of the Czech Republic at four different locations: Velké Pavlovice, Kvítkovice, Prakšice and Horní Suchá.

**Figure 3:**
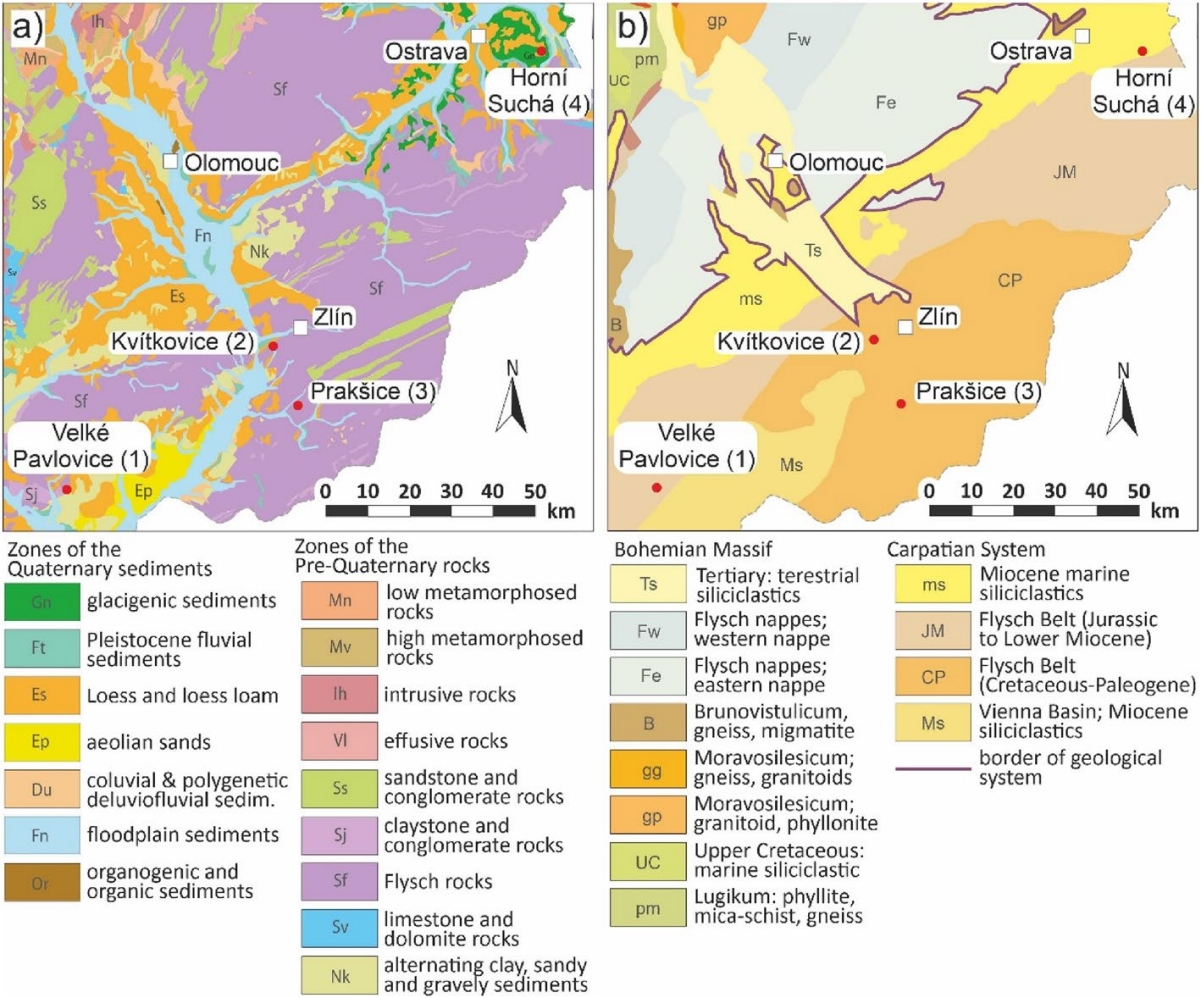
Maps of the area of interest, (**a**) map of engineering-geological zones, (**b**) map of geological division. Schematic figures made by the authors using CorelDRAW Graphic Suite 2019 software www.coreldraw.com.

As for engineering-geology (Fig. 3a), in locality 1 in Velké Pavlovice, and locality 2 in Kvítkovice, the four interest localities of the waste landfills are in the zone of alternating clay, sandy and gravely sediments. In locality 3 in Prakšice it is the zone of flysch rocks, and the locality 4 in Horní Suchá is characteristic of the zone of loess and loess loam.

From the geological point of view (Fig. 3b), the interest localities are situated in the Western Carpathians. The PreQuaternary rocks of the locality 1 are found in Miocene marine sediments covering the Flysch Belt (Jurassic to Lower Miocene). The PreQuaternary rocks of the localities 2 and 3 are found in the Rača Unit, the Magura Group of the Flysch Belt Nappe. The PreQuaternary rocks of the locality 4 are located in the Miocene marine sediments.

**Figure 4:**
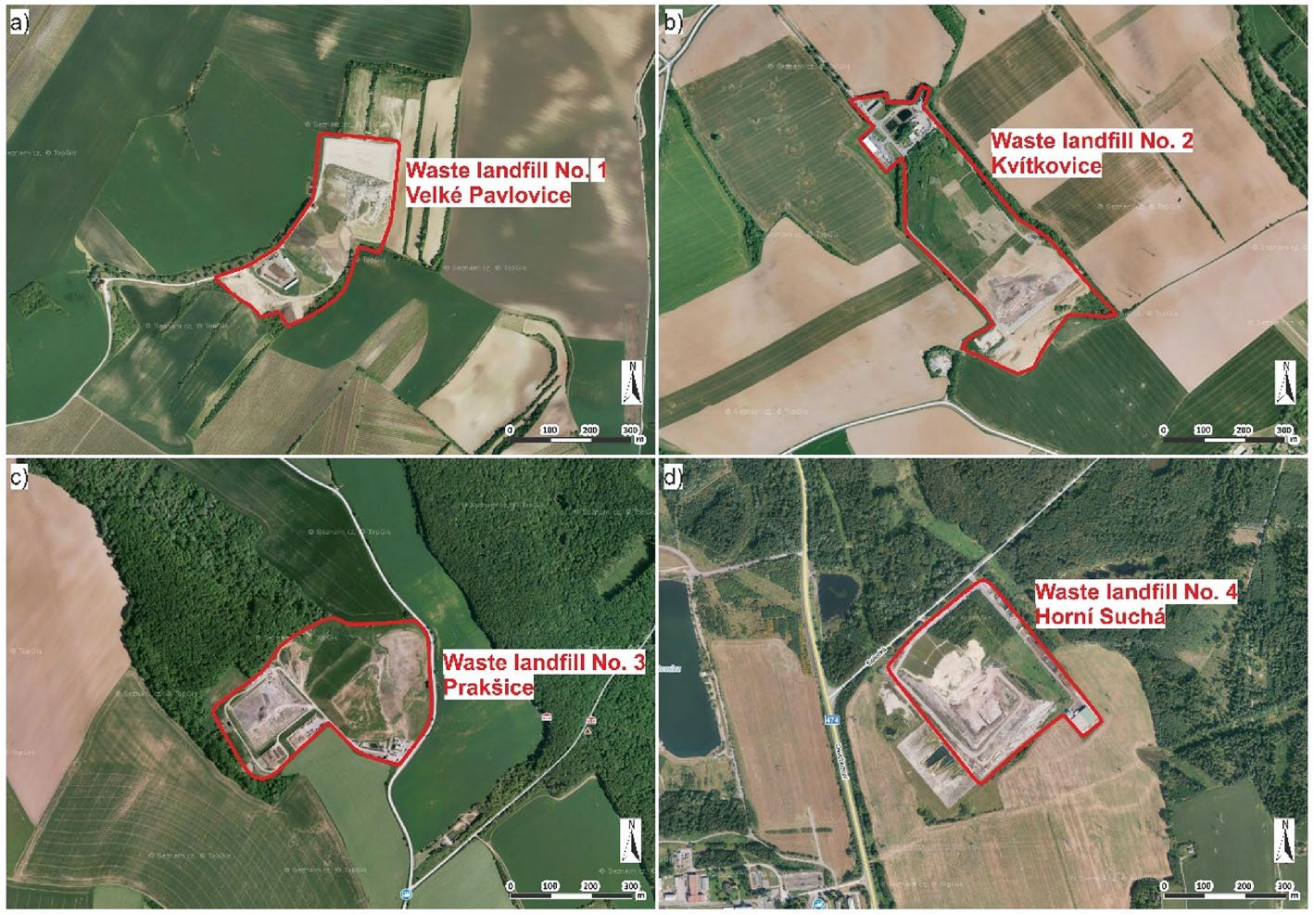
Interest localities of the four waste landfills, (**a**) aerial photo of waste landfill 1 in Velké Pavlovice, (**b**) aerial photo of waste landfill 2 in Kvítkovice, (**c**) aerial photo of waste landfill 3 in Prakšice, and (**d**) aerial photo of waste landfill 4 in Horní Suchá; Aerial photographs obtained with permission from www.seznam.cz.

Waste landfill 1 is located in Velké Pavlovice, South-Moravian Region, and has an area of 9.7 ha (Fig. 4a). Waste landfill 2 is in Kvítkovice, Zlín Region, and has an area of 18.2 ha (Fig. 4b). Waste landfill 3 is located in Prakšice, Zlín Region, and covers an area of 4.8 ha (Fig. 4c). Waste landfill 4 is in Horní Suchá, Moravia-Silesian Region and has an area of 14.1 ha (Fig. 4d). All analysed localities were in category 3 (Model 1) and category 2 (Model 2).

## Methodology

**Figure 5:**
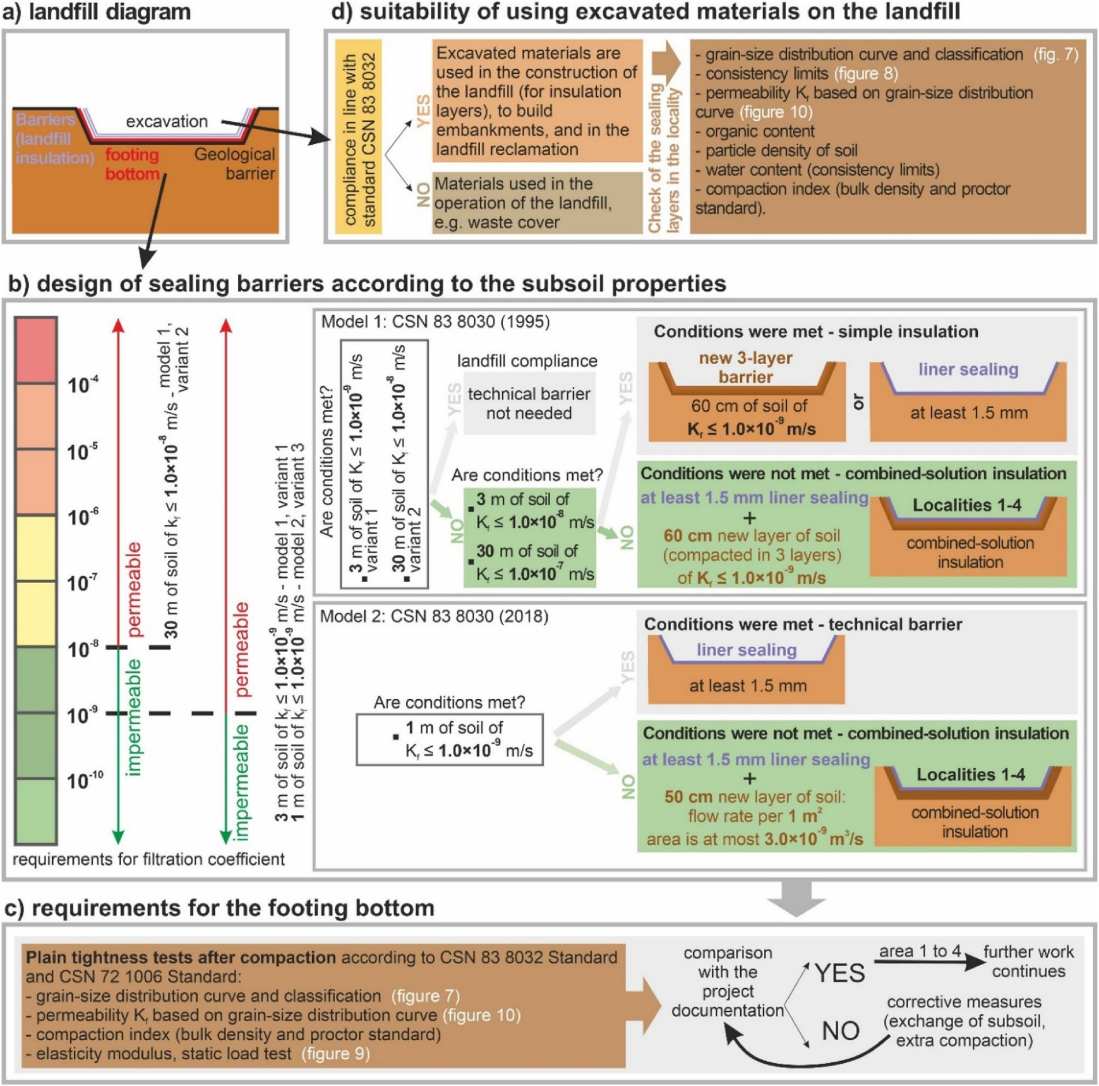
Requirements and research aim, (**a**) landfill diagram, (**b**) design of sealing barriers according to the subsoil properties, (**c**) requirements for the footing bottom, (**d**) suitability of using excavated materials on the landfill.

The paper reports 4 case studies investigating waste landfills. Each locality was assessed for the geological barrier in the landfill’s *footing bottom*. Moreover, we also focused on the issue of earthwork (Fig. 5a).

Figure 5 shows the requirements and research objectives of this research. The graphic shows a diagram of the landfill, the design of the sealing barriers in relation to the properties of the soil, the requirements for the footing bottom and the suitability of applying the excavated material to the landfill.

If we evaluate the requirements for the *design of sealing barriers based on the properties of the geological subsoil*, we must first assess the properties of the soil massif below the footing bottom using engineering-geological investigations (Fig. 5b). All the four case studies fall in Category 3 (Model 1) and Category 2 (Model 2). For this reason, Fig. 3b gives a detailed geological barrier evaluation related to the given 4 case studies only. The other categories are described and compared above—the waste landfill categories are described in Fig. 2 and the four case studies are marked in the red box. The decision-making flowchart for the landfill sealing solutions in connection with the 4 case studies (based on the models and their categories) is shown in Fig. 5b on the right. The green colour designates the sequence of selecting a landfill sealing solution. On the left, there is an impermeability classification based on filtration coefficient related to models 1 and 2 and their categories. The requirements and important boundaries are shown graphically. The first boundary is determined by the filtration coefficient lower than or equal to 1.0 * 10^–8^ m/s (category 3 of Model 1). The second boundary is determined by the filtration coefficient lower than or equal to 1.0 * 10^–9^ m/s, where investigations are carried out as deep as 3 m in Model 1 (category 3) and as deep as 1 m in Model 2 (category 2).

All the values above the limit boundary mean that the environment is permeable (negative variant—marked in red). On the contrary, the opposite variant means that the environment is impermeable, which is a positive (desirable) variant (marked in green).

As for the *requirements for the footing bottom* (Fig. 5c), discussed in connection with tightness tests post compaction in line with CSN 83 8032^52^ Standard and CSN 72 1006^53^ Standard, it is important to implement a grain-size distribution curve for the landfill subsoil materials and classify these as foundation soils. Next, it is important to determine the filtration coefficient, degree of compaction, bulk density, proctor standard test, modulus of elasticity (statical load test). In case the soil materials correspond to the desired properties, work continues without any corrective measures, e.g., exchange of subsoil, extra compaction, etc.

As for the evaluation of the *excavated materials* (Fig. 5d), we can either use these in the construction of the landfill when establishing the sealing layer, embankments, or in the reclamation of the landfill (when waste disposal is terminated). If the requirements are not met, the materials may be used to cover waste during landfill operation. For this purpose, subsequent laboratory tests of excavated materials are implemented, namely grain-size distribution curve and classification, consistency limits, permeability, filtration coefficient, organic content, particle density of soil, moisture, compaction index (bulk density and proctor standard).

*Permeability,* or *impermeability,* of the geological environment may be evaluated using different methods in engineering geology. The most important *assessment criterion* is the *purpose* the geological environment is supposed to have in the engineering structure.

**Figure 6:**
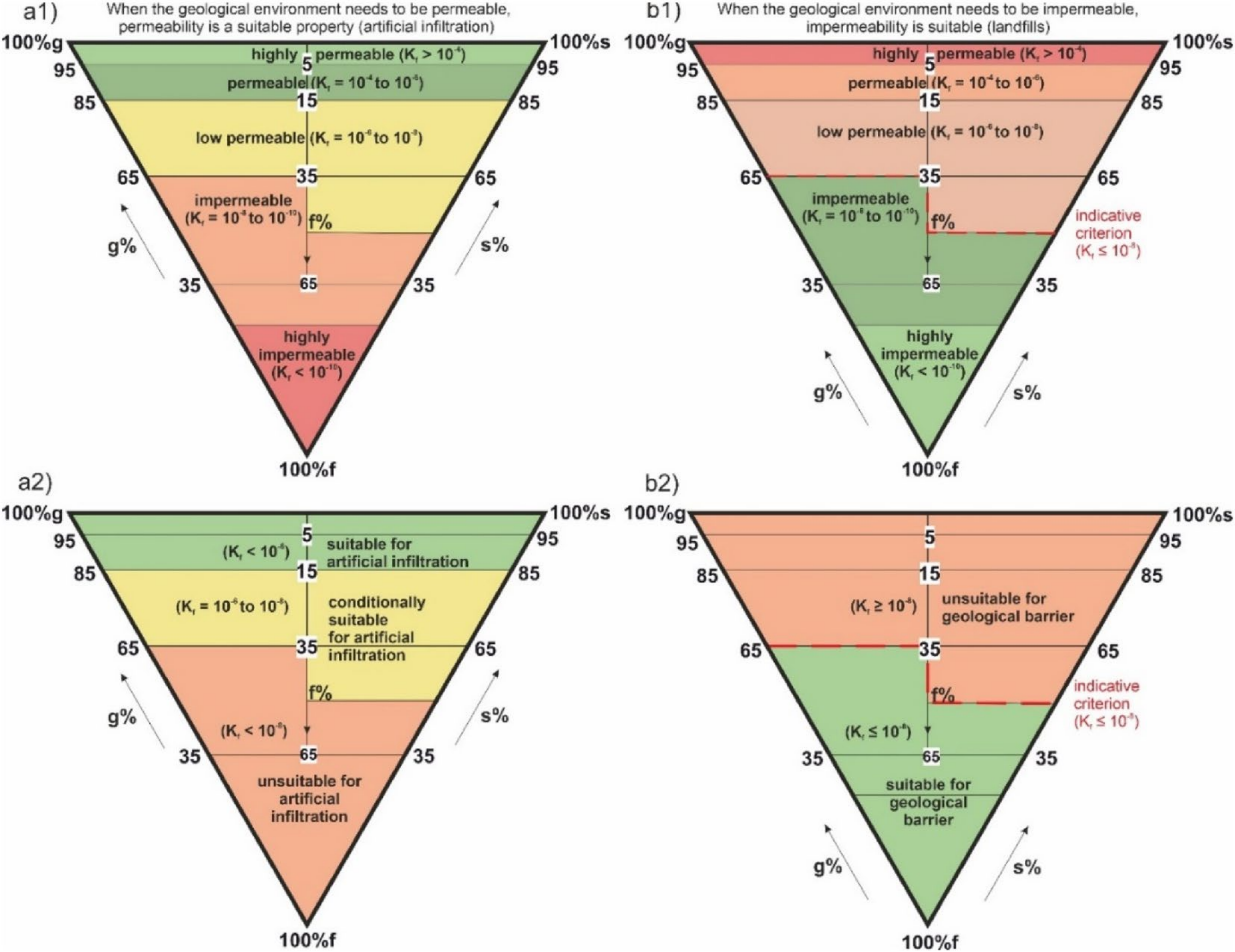
Reference classification triangles of permeability and impermeability of the geological environment related to sandy, gravel and fine-grained soils (foundation soils), (**a1**) classification triangle of soil permeability (based on filtration coefficient), (**a2**) classification triangle of soil suitability for artificial infiltration, (**b1**) classification triangle of impermeability, (**b2**) classification triangle of soil suitability for the geological barrier (example for Model 1, waste landfill categories 2 and 3).

Figure 6 presents the permeability and impermeability reference classification triangles of the geological environment associated with sandy, gravel and fine-grained soils (foundation soils). Triangle a1) shows the classification of the permeability of the soil (based on filtration coefficient). Figure 6a2) shows the soil suitability classification triangle for artificial infiltration. Figure 6b1) shows the impermeability classification triangle. The last diagram b2) gives information on the soil suitability classification triangle for the geological barrier (example for Model 1, waste landfill category 2 and 3).

At times the permeable environment may be perceived as a positive property (Fig. 6a1), e.g., when we deal with the tasks of artificial infiltration (Heviankova et al. 2018) in the period of droughts. Artificial infiltration is directly related permeability and grain-size distribution of soils (Zięba 2017). In case of artificial infiltration, we may redirect water from rivers or ponds into the ground water reservoirs that are drying out. Such water is not supplied into the reserves directly but must be filtered through the geological environment to improve its properties. This way, water is filtrated thanks to its migration in the geological environment and the porous environment (e.g., gravel-sand) may help improve the water properties. There are more examples when the permeability is perceived in a positive manner in engineering geology and/or hydrogeology. Another example is the situation when we need fast drainage (Turček et al. 2005) of the geological environment and more permeable soils lead to easier and cheaper solutions of drainage projects.

An example of *soil classification* where permeability of the geological environment is perceived *positively* is shown in Fig. 6a1. The most permeable soils (Heviankova et al. 2018) are gravels and sands marked in green (*suitable* for artificial infiltration, Fig. 6a2). On the contrary, impermeable environment which does not allow water passage into lower layers is marked in red (*unsuitable* for the purpose*)*.

On the other hand, permeable geological environment may be problematic, and thus *impermeability becomes a positive property*. Among such examples are the need for geological barriers (discussed herein) when establishing waste landfills, as such barriers represent natural insulation against the spread of potential contamination. *Reference soil classification* related to the geological barrier (environment) impermeability of landfills is given in Fig. 6b1. This classification is of an indicative value only because there are also other properties that need to be considered, besides the grain-size analysis (marking gravel, sandy and fine-grained soils). However, this reference classification (the classification triangle of foundation soils) is very important for engineering geologist and geotechnicians.

Contrary to the infiltration example above, this triangle of permeability (un)suitability (Fig. 6b2) has a reverse character (Fig. 6a1) here. Impermeable fine-grained soils are marked in green (Fig. 6b2) because as subsoils they are perceived as a positive geological barrier. The red colour is used to mark permeable gravel soils, in which contamination would spread. The triangle is shown for waste landfill category II (investigations as deep as 1 m of soil) and category III (investigations as deep as 3 m of soil) of Model 1, where the suitability or unsuitability is decided based on the filtration coefficient lower than or equal to 1.0 * 10^–8^ m/s. The suitable parameters for the geological environment are thus below this value (green colour) and the red colour marks unsuitable environments above the value. The evaluation based on the filtration coefficient is only one of the criteria for the geological barrier. In order for the geological barrier to meet the landfill category parameters, it must also correspond to the geometry requirements for the depth of the earthwork which verifies the impermeability in the geological environment.

## Results of physical characteristics and classification

The research presented here was carried out for all four sites (Velké Pavlovice, Kvítkovice, Prakšice and Horní Suchá). Therefore, it is not necessary to divide the results of the soil classification and its basic properties into two parts.

**Figure 7:**
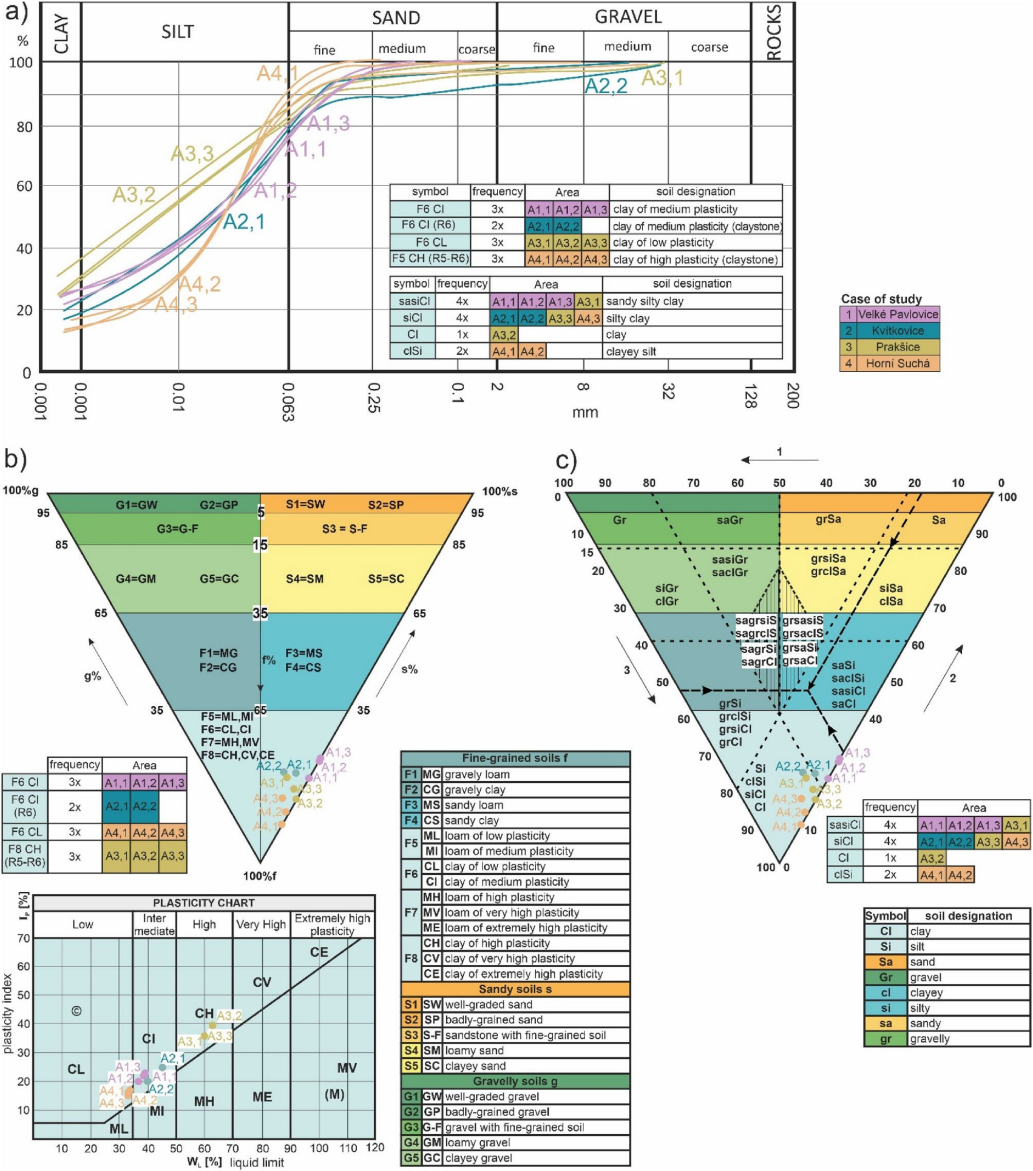
Classification of the case studies according to the foundation soil classification, (**a**) grain-size distribution curve, (**b**) classification triangle of foundation soils based on CSN 73 1001, (**c**) classification triangle of foundation soils based on CSN EN ISO 14,688–2 (72 1003).

In the locality 1 in Velké Pavlovice we took 3 samples from the footing bottom for the grain-size analysis (Fig. 7a). According to the first classification of foundation soils (^51^, Fig. 7b), all these had the character of clay of medium plasticity (F6 CI). According to the second classification^54^, Fig. 7c), they had the character of sandy silty clay (sasiCl).

In the locality 2 in Kvítkovice, 2 samples were drawn from the footing bottom (Fig. 7a). They had the character of clay of medium plasticity according to the first classification (^51^, Fig. 7b), and are clay eluvium formed by severe weathering based on the classification triangle of foundation soils^51^. In terms of the second classification (^54^, Fig. 7c) the samples have the character of silty clay (siCl) based on the classification triangle of foundations soils.

Three samples were drawn (Fig. 7a) in the locality 3 in Prakšice. According to the first classification (Fig. 7b), they had the character of clay of low plasticity (F6 CL). According to the second classification (Fig. 7c), the first sample was clay (Cl), the second was characterized as sandy silty clay (sasiCl), and the third as silty clay (siCl).

In the locality 4 in Horní Suchá, we sampled 3 samples (Fig. 7a). According to the first classification (Fig. 7b) it was clay of high plasticity (F5 CH), formed by clay weathering. According to the second classification (Fig. 7c), the 2 soil samples had the character of clayey silt (clSi) and the third had the character of silty clay (siCl).

**Figure 8:**
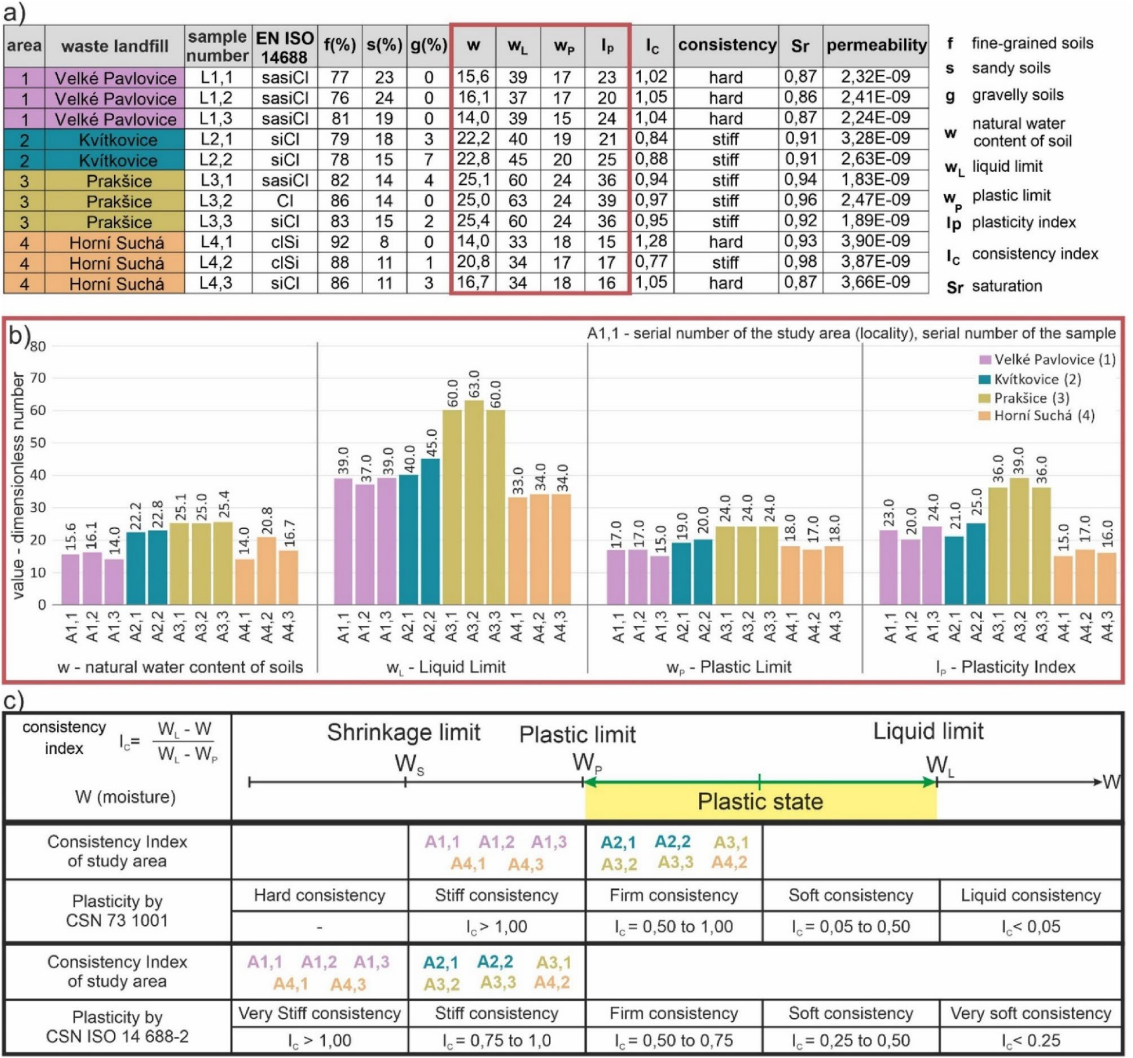
Selected soil properties in the case study localities 1, 2, 3 and 4 (**a**) table of soil properties, (**b**) charts with water content, liquid limit, plastic limit and plasticity index (**c**) classification of the localities in terms of consistencies.

With regard to the fact that all the localities contained fine-grained soils, we had to determine the most decisive properties (Fig. 8a,b) that influence engineering-geological evaluations to the greatest extent. These properties were determined in the laboratory from the bore-hole samples.

The first such property was *natural water content (w)* with an identified range of 14.0–25.4. In the locality 1 in Velké Pavlovice the range was 14.0–6.1. In the locality 2 in Kvítkovice it was 22.2–22.8., in the locality 3 in Prakšice there were the highest values (25.0–25.4), and in the locality 4 in Horní Suchá the range was 14.0–20.8.

*Liquid limit (wL)* was determined by a cone penetration test. The determined value ranges were 33.0–63.0. The lowest values were in the locality 4 (33.0–34.0). On the contrary, the highest values were in the locality 3, where the range was 60.0–63.0. In the locality 1 the range was 37.0–39.0, and in the locality 2 it was 40.0–45.0.

The third property was *plastic limit (wp)*, which was identified as 15.0–24.0. The lowest values were observed in the locality 1 (15.0–17.0). The highest values were in the locality 3 (24.0). The locality 2 had the range of 19.0–20.0, and the locality 4 had the range of 17.0–18.0.

The last determined property was *plasticity index (Ip)* measured from 15.0 to 39.0. In the locality 4 there were the lowest values (15.0–17.0). On the contrary, the top plasticity index was observed in the locality 3 (36.0–39.0). In the locality 1 the values were 20.0–24.0, and in the locality 2 it was 21.0–25.0.

As for the consistencies in the fine-grained soil footing bottom, we observed only two consistencies: hard (A1,1; A1,2; A1,3; A4,1; A4,3) and stiff (A2,1; A2,2; A3,1; A3,2; A3,3; A4,2) (Fig. 8c).

## Results of geological barrier evaluation

**Figure 9:**
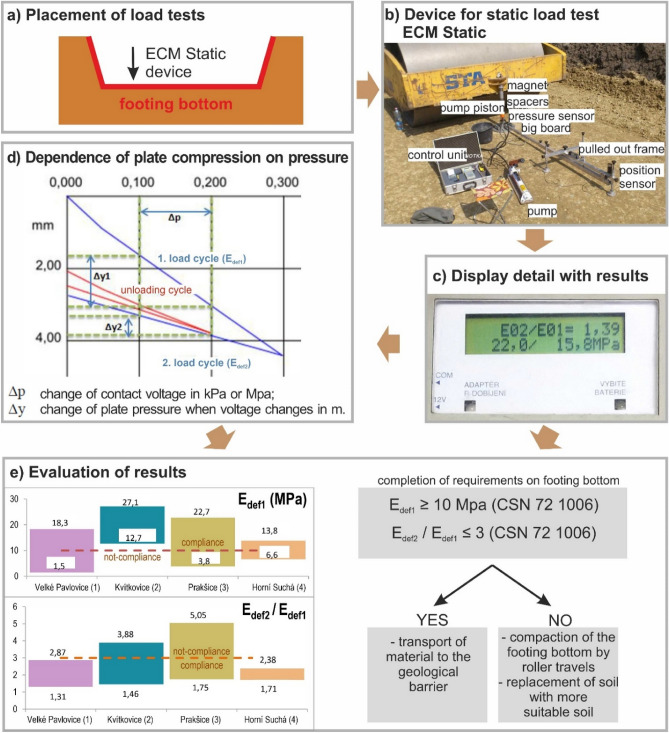
Soil compressibility of case study localities 1, 2, 3 and 4 (**a**) placement of load tests, (**b**) device for static load test ECM Static, (**c**) display detail with results, (**d**) dependence of plate compression on pressure, (**e**) Evaluation of results.

If we deal with the issue of geological barrier, which is primarily focused on impermeability, we need more parameters considering load-bearing capacity and settlement. To measure *compressibility* using the static pile load test, we selected a site on the landfill’s footing bottom (Fig. 9a). ECM-Static was used for measurements (Fig. 9b) and sufficient counterweight using a roller must be used for measurements. The device consists of a pressure sensor with a pump. Compressive force is transferred onto a plate and its movement is recorded by sensors. The sensors of position and pressure send the values to the control unit, which manages the test and shows results (Fig. 9c). The load test plate was positioned on a consolidated surface and small unevenness was levelled off. If hollows remained under the plate, these were filled with fine sand. The load test was carried out in two cycles under different load degrees and relief degree. The resulting values are elasticity modulus E_def1_ from the first cycle, and E_def2_ from the second cycle (Fig. 9d).

Two assessment criteria influencing the settlement were chosen for the four landfills in terms of subsoil compressibility. The first criterion was that the elasticity modulus from the first cycle should be over or equal to 10 MPa (E_def1_ ≥ 10 MPa) based on CSN 72 1006 Standard (1998, Fig. 9e). Only the locality 2 in Kvítkovice complied with this criterion as the elasticity modulus was 12.7–27.1 MPa (Fig. 9e). In the other three localities, we found elasticity modulus of 1.5–18.3 MPa in locality 1 in Velké Pavlovice, 3.8–22.7 MPa in the locality 3 in Prakšice, and 6.6–13.8 MPa in the locality 4 in Horní Suchá. These values imply that the criterion set in the standard was not met. The second assessment criterion was the ratio of the elasticity modulus from the first and second load cycle (E_def2_ / E_def1_) to be higher than or equal to 3 based on CSN 72 1006 Standard^53^. Only two localities met this criterion, namely locality 1 in Velké Pavlovice (E_def2_ / E_def1_ from 1.31 to 2.87) (Fig. 9e) and locality 4 in Horní Suchá (1.71–2.38). On the contrary, this criterion was not met in the locality 2 in Kvítkovice (1.46–3.88) or locality 3 in Prakšice (1.75–5.05). Because none of the localities met both the criteria, subsoil had to be compacted using a 15-ton roller to ensure better compactness of the footing bottom.

**Figure 10:**
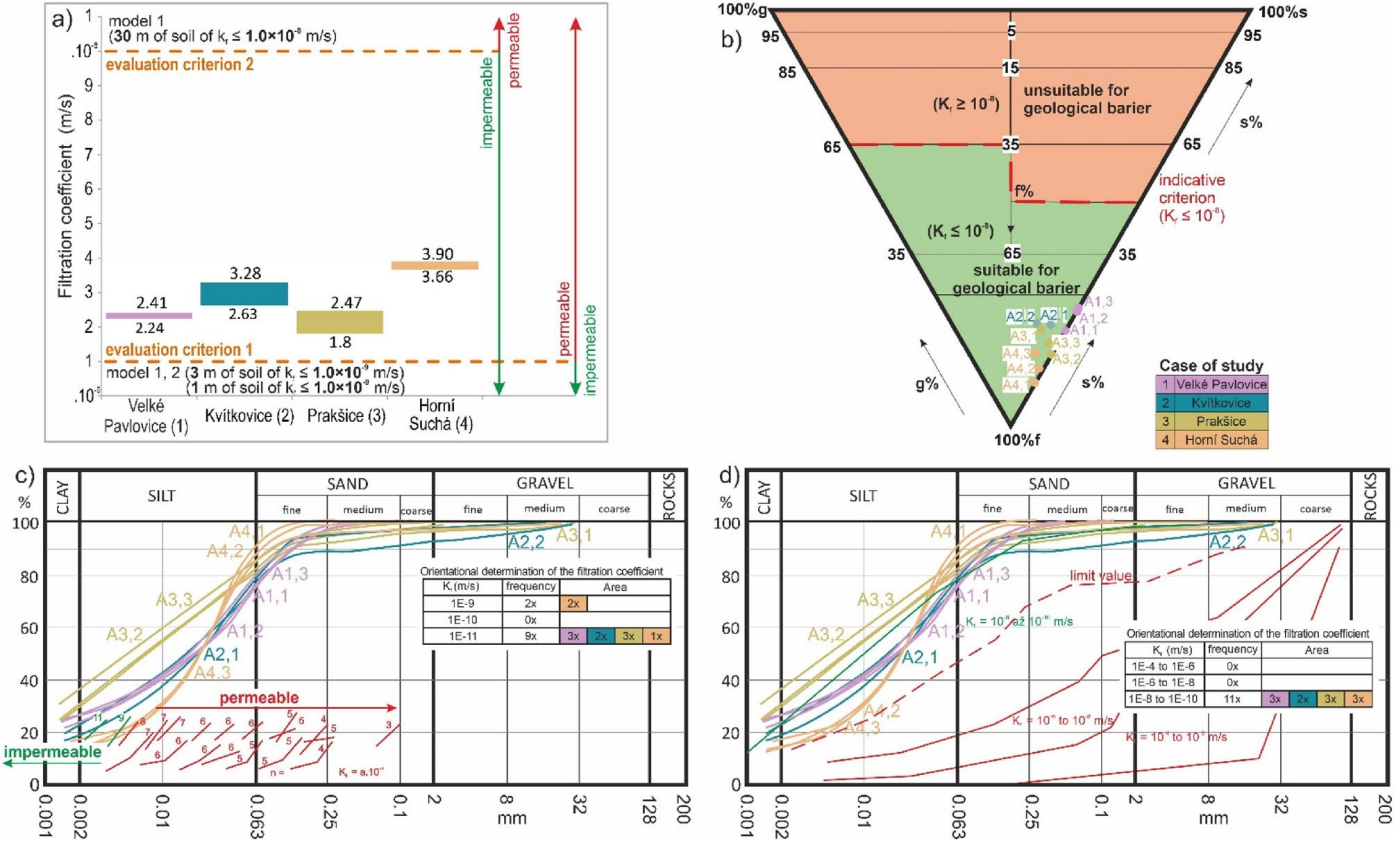
(**a**) Filtration coefficient in four localities 1, 2, 3 and 4, (**b**) classification triangle of soil suitability for the geological barrier (example for Model 1, landfill categories 2 and 3), (**c**) indicative value of permeability based on grain-size distribution curve (**d**) indicative filtration coefficient based on grain-size distribution curve using permeability curves.

If we evaluate the *filtration coefficient* in the localities, we find an interval of 2.24 * 10^–9^–2.41 * 10^–9^ m/s in the locality 1 in Velké Pavlovice; 2.63 * 10^–9^–3.28 * 10^–9^ m/s in the locality 2 in Kvítkovice; 1.8 * 10^–9^–2.47 * 10^–9^ m/s in the locality 3 in Prakšice; and 3.66 * 10^–9^–3.90 * 10^–9^ m/s in the locality 4 in Horní Suchá (Fig. 10).

Two permeability criteria were used in the landfills. The first is the limit value of the soil material to be lower than or equal to 1.0 * 10^–9^ m/s. None of the localities met the limit. The second limit value must be lower than or equal to 1.0 * 10^–8^ m/s. All 4 localities met this assessment criterion.

**Figure 11:**
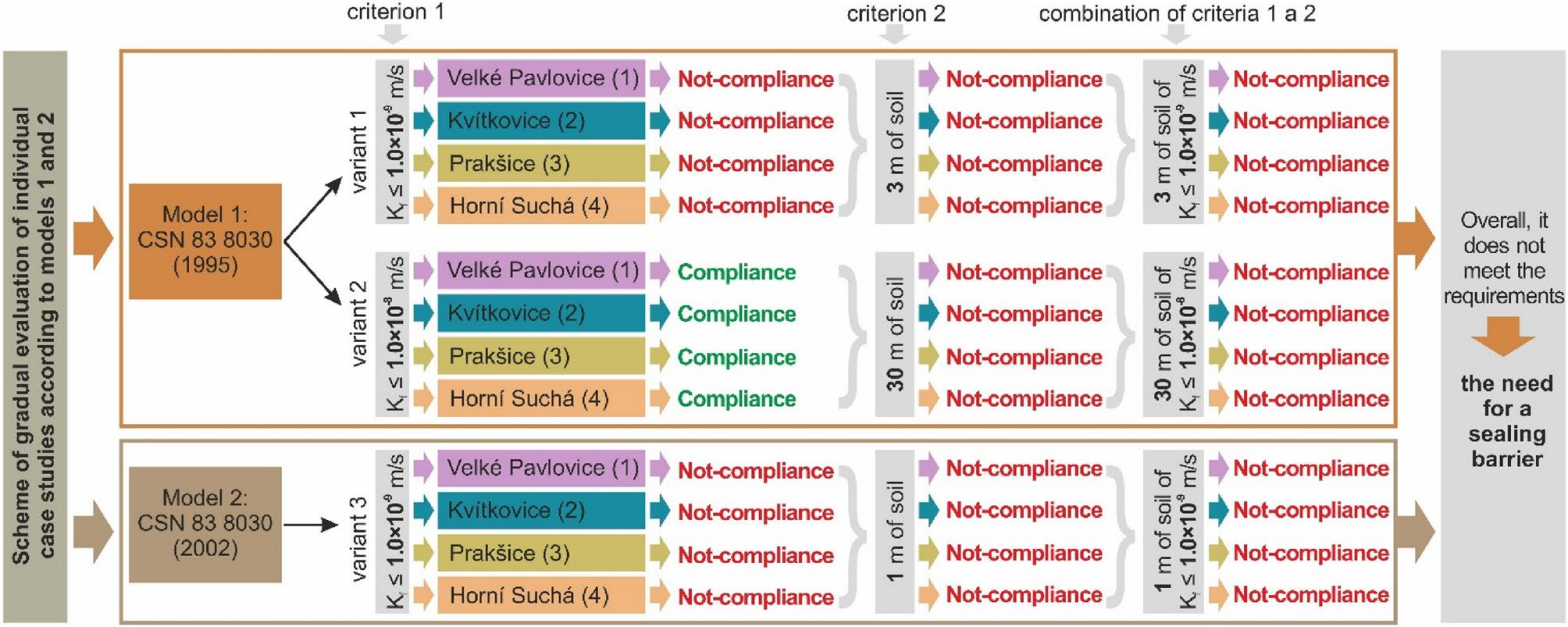
Diagram of assessment criteria to evaluate the tightness of the geological environment (natural geological barrier) for the purposes of establishing landfills in 4 localities (no. 1 Velké Pavlovice, no. 2 Kvítkovice, no. 3 Prakšice and no. 4 Horní Suchá) and final assessment (all 4 case studies do not meet the criteria and an anthropogenic barrier must be installed).

The *evaluation of the natural geological barrier* (Fig. 11) shows the evaluation of all the case studies based on assessing the impermeability of the geological subsoil below the landfill. All the localities were assessed based on the two models arising from the standards. The diagram shows all three assessment criteria.

*The first assessment criterion* was permeability based on *filtration coefficient*. It shows that in the first model (variant 1, Fig. 11) none of the localities meet the criterion of filtration coefficient K_f_ ≤ 1.0 * 10^–9^ m/s. However, in the variant 2 (Fig. 11) based on a different filtration coefficient K_f_ ≤ 1.0 * 10^–8^ m/s, all the four localities meet the value. This lower value is compensated for by another criterion which is stricter than in variant 1 (see below). As for Model 2 (variant 3, Fig. 11), none of the localities met the filtration coefficient K_f_ ≤ 1.0 * 10^–9^ m/s either.

*The second assessment criterion* is *geometry* related to the impermeability at a certain subsoil depth. This means that we evaluate the geological environment below the footing bottom. At the same time, none of the parts of the geological environment may have weaker impermeability. In variant 1, we assess impermeability of the geological environment at the depth of 3 m below the footing bottom. This criterion was not met by any of the localities. In variant 2, there is a requirement for impermeability as deep as 30 m, which was not met by any locality. In variant 3, impermeability is measured as deep as 1 m, which was not complied with at any of the case studies.

*The third assessment criterion* was the *combination of criteria 1 and 2*. This means when the results were positive in both the criteria, the combined criterion is also positive (compliance). On the contrary, when either one or both results are negative (non-compliance), the third (combined) criterion is also negative. This occurred in all four assessed localities. This implies that the requirements for the natural geological barrier were not met and thus it is vital to install an anthropogenic (technical) barrier (liner) in all the 4 localities which must meet the geometric-technical and material requirements of the model (standards) in the relevant variants—see Fig. 2.

## Conclusion

Having implemented this comparative engineering-geology analysis of 4 landfill case studies, we must state that the impermeable subsoil poses a certain disadvantage, but it also represents an important specific condition in landfills. It is strategic in the sense that if the landfill is not built on a sufficiently impermeable subsoil (defined by filtration coefficient) of a sufficient thickness of the geological environment (defined by depth in metres), the financial costs to establish the landfill are higher because the solution cannot take advantage of a natural insulation of a geological barrier to prevent potential contamination in waste landfills. This way, high-quality impermeable subsoil is a boundary condition for landfills. From the engineering-geological point of view, when investigating such localities, besides the load-bearing capacity and settlement, we need to consider the impermeability of the geological environment.

We carried out four case studies in the localities of Velké Pavlovice (filtration coefficient of 2.24 * 10^–9^–2.41 * 10^–9^ m/s), Kvítkovice (2.63 * 10^–9^–3.28 * 10^–9^ m/s), Prakšice (1.80.10^–9^–2.47 * 10^–9^ m/s) and Horní Suchá (3.66 * 10^–9^–3.90 * 10^–9^ m/s). The results show that the required limit value (1.0 * 10^–8^ m/s) is met only for variant 2 of Model 1 (only 1 of the 3 studied variants) in the localities. The other 2 variants (variants 1 and 3) do not meet the limit value of 1.0 * 10^–9^ m/s. All the 3 studied variants do not meet the second criterion, which is impermeability of subsoil at certain depth (in variant 1 it is 3 m, and in variant 2 it is 30 m, and in variant 3 it is 1 m). This concerns all of the four localities, i.e., Pavlovice (case study 1), Kvítkovice (case study 2), Prakšice (case study 3) and Horní Suchá (case study 4).

We can approach landfill impermeability assessment in a number of ways. In the study, we used 2 methodologies (models) in 3 variants (the first model had 2 variants, and the second model had 1 variant). The first variant is the natural geological barrier of 3 m and compliance with filtration coefficient lower than 1.0 * 10^–9^ m/s. The second variant is impermeable geological subsoil of 30 m at the filtration coefficient lower than 1.0 * 10^–8^ m/s. The third variant is characterized by impermeable geological environment of 1 m and filtration coefficient lower than 1.0 * 10^–9^ m/s.

The examined localities did not meet the boundary conditions in any of the variants, and thus technical insulation barrier had to be installed—see Sect. 2 and Fig. 2.

If we evaluate the approach to engineering-geological investigations of landfills based on the waste hazard character, the rule is: the more hazardous waste, the more demanding requirements for the depth of the engineering-geological investigations. If the required criteria for the natural barrier depth and filtration coefficient are not met, the natural geological barrier is supplemented by a technical insulation barrier. In variant 2, the maximum depth is 30 m, while in variant 3 it is only 1 m (variant 1—depth of 3 m). This means that variants 1 and 3 are less strict than variant 2 considering the depth of engineering-geological investigations. However, lower depths of investigations mean stricter requirements for impermeability of the geological environment assessed based on the filtration coefficient. Variant 2 had a less strict criterion K_f_ ≤ 1.0 * 10^–8^ m/s when compared with variants 1 and 3 (K_f_ ≤ 1.0 * 10^–9^ m/s). The more hazardous waste, the higher demands on the impermeability of the geological environment applied by filtration coefficient combined with the required depth of impermeable subsoil.

## Data Availability

All data analysed during this study are included in this published article.

## References

[1] Ersoy H, Bulut F, Berkün M (2013). Landfill site requirements on the rock environment: A case study. Eng. Geol..

[2] Huang Y, Fan G (2016). Engineering geological analysis of municipal solid waste landfill stability. Nat. Hazards.

[3] Israde-Alcantara I, Delgado OB, Chavez AC (2005). Geological characterization and environmental implications of the placement of the Morelia dump, Michoacán, central Mexico. J. Air Waste Manag. Assoc..

[4] Xiang R, Xu Y, Liu YQ, Lei GY, Liu JC, Huang QF (2019). Isolation distance between municipal solid waste landfills and drinking water wells for bacteria attenuation and safe drinking. Sci. Rep..

[5] Bouazza, A. & Kavazanjian Jr, E. Construction on former landfills. In Proceedings 2nd ANZ Conference on Environmental Geotechnics, Newcastle (pp. 467–482) (2004).

[6] Li L, Li J, Huang J, Liu H, Cassidy MJ (2017). The bearing capacity of spudcan foundations under combined loading in spatially variable soils. Eng. Geol..

[7] Ma P, Ke H, Lan J, Chen Y, He H (2019). Field measurement of pore pressures and liquid-gas distribution using drilling and ERT in a high food waste content MSW landfill in Guangzhou, China. Eng. Geol..

[8] Schneider P, Oswald KD, Weiß B, Littmann R (2017). Assessing geotechnical risks in the frame of landfill engineering in eastern Europe. J. Geol. Resource Eng..

[9] Tunusluoglu MC (2020). Engineering geological assessment and determination of bearing capacity of the Buyukyenice dam site (Balıkesir, Turkey). Arab. J. Geosci..

[10] Yao YP, Qi SJ, Che LW, Chen J, Han LM, Ma XY (2018). Postconstruction settlement prediction of high embankment of silty clay at Chengde airport based on one-dimensional creep analytical method: Case study. Int. J. Geomech..

[11] Han Z, Ma H, Shi G, He L, Wei L, Shi Q (2016). A review of groundwater contamination near municipal solid waste landfill sites in China. Sci. Total Environ..

[12] Li Y, Li J, Chen S, Diao W (2012). Establishing indices for groundwater contamination risk assessment in the vicinity of hazardous waste landfills in China. Environ. Pollut..

[13] Mepaiyeda S, Madi K, Gwavava O, Baiyegunhi C (2020). Geological and geophysical assessment of groundwater contamination at the Roundhill landfill site, Berlin, Eastern Cape, South Africa. Heliyon.

[14] Rapti-Caputo D, Sdao F, Masi S (2006). Pollution risk assessment based on hydrogeological data and management of solid waste landfills. Eng. Geol..

[15] Baltrūnas V, Slavinskienė G, Karmaza B, Pukelytė V (2020). Effectiveness of a modern landfill liner system in controlling groundwater quality of an open hydrogeological system, SE Lithuania. J. Environ. Eng. Landsc. Manag..

[16] Dang M, Chai J, Xu Z, Qin Y, Cao J, Liu F (2020). Soil water characteristic curve test and saturated-unsaturated seepage analysis in Jiangcungou municipal solid waste landfill, China. Eng. Geol..

[17] Mishra S, Tiwary D, Ohri A, Agnihotri AK (2019). Impact of Municipal Solid Waste Landfill leachate on groundwater quality in Varanasi, India. Groundwater Sustain. Dev..

[18] Przydatek G, Kanownik W (2019). Impact of small municipal solid waste landfill on groundwater quality. Environ. Monit. Assess..

[19] Vahabian M, Hassanzadeh Y, Marofi S (2019). Assessment of landfill leachate in semi-arid climate and its impact on the groundwater quality case study: Hamedan, Iran. Environ. Monit. Assessm..

[20] Aksoy E, San BT (2019). Geographical information systems (GIS) and multi-criteria decision analysis (MCDA) integration for sustainable landfill site selection considering dynamic data source. Bull. Eng. Geol. Environ..

[21] Dzhamalov RG, Medovar YA, Yushmanov IO (2019). Principles of MSW landfill sites’ placement depending on geological and hydrogeological conditions of territories (based on Moscow region). Water Resour..

[22] El Maguiri A, Kissi B, Idrissi L, Souabi S (2016). Landfill site selection using GIS, remote sensing and multicriteria decision analysis: Case of the city of Mohammedia, Morocco. Bull. Eng. Geol. Env..

[23] Khodaparast M, Rajabi AM, Edalat A (2018). Municipal solid waste landfill siting by using GIS and analytical hierarchy process (AHP): A case study in Qom city, Iran. Environ. Earth Sci..

[24] Yildirim V, Memisoglu T, Bediroglu S, Colak HE (2018). Municipal solid waste landfill site selection using multi-criteria decision making and GIS: Case study of Bursa province. J. Environ. Eng. Landsc. Manag..

[25] Al Farishi B, Setiawan MR (2019). The mapping of contamination potential surrounding bakung landfill based on geological studies. IOP Conf. Ser. Earth Environ. Sci..

[26] Aleisa E, Al-Jarallah R, Shehada D (2019). The effect of geological and meteorological conditions on municipal waste management systems: A life cycle assessment approach. Int. J. Environ. Sci. Technol..

[27] Høyer AS, Klint KES, Fiandaca G, Maurya PK, Christiansen AV, Balbarini N, Bjerg PL, Hansen TB, Møller I (2019). Development of a high-resolution 3D geological model for landfill leachate risk assessment. Eng. Geol..

[28] Huyer AS, Klint KES, Fiandaca G, Maurya PK, Christiansen AV, Balbarini N, Bjerg PL, Hansen TB, Muller I (2019). Development of a high-resolution 3D geological model for landfill leachate risk assessment. Eng. Geol..

[29] Jafari K, Bajestani AM, Moghaddas NH, Ghazi A (2017). Landfill siting for municipal waste: A case study in Ardebil. J. Eng. Geol..

[30] Leton TG, Omotosho O (2004). Landfill operations in the Niger delta region of Nigeria. Eng. Geol..

[31] Baawain MS, Al-Futaisi AM, Ebrahimi A, Omidvarborna H (2018). Characterizing leachate contamination in a landfill site using Time Domain Electromagnetic (TDEM) imaging. J. Appl. Geophys..

[32] Dumont G, Robert T, Marck N, Nguyen F (2017). Assessment of multiple geophysical techniques for the characterization of municipal waste deposit sites. J. Appl. Geophys..

[33] Saatsaz M, Monsef I, Rahmani M, Ghods A (2018). Site suitability evaluation of an old operating landfill using AHP and GIS techniques and integrated hydrogeological and geophysical surveys. Environ. Monit. Assess..

[34] Karagüzel R, Kilic R (2000). The effect of the alteration degree of ophiolitic melange on permeability and grouting. Eng. Geol..

[35] Marschalko M, Putiška R, Yilmaz I, Niemiec D, Vybíral V, Popielarczyk D, Matuszková B (2020). Investigation of a hazardous uncontrolled dumpsite in an oxbow lake of the Nitra River for pollution potential: A case study. Environ. Monit. Assess..

[36] Marschalko M, Vicherek P, Vicherková M, Yilmaz I, Kubáč J, Popielarczyk D, Kempa T, Yang S (2020). Soil contamination by tar in the alluvial sediments: Case study of the brownfield remediation project in the Czech Republic. Environ. Earth Sci..

[37] Srivastava A, Babu GS, Haldar S (2010). Influence of spatial variability of permeability property on steady state seepage flow and slope stability analysis. Eng. Geol..

[38] Yilmaz I, Marschalko M, Bednarik M, Kaynar O, Fojtova L (2012). Neural computing models for prediction of permeability coefficient of coarse-grained soils. Neural Comput. Appl..

[39] Yang R, Xu Z, Chai J, Qin Y, Li Y (2016). Permeability test and slope stability analysis of municipal solid waste in Jiangcungou Landfill, Shaanxi, China. J. Air Waste Manag. Assoc..

[40] Yang, R., Xu, Z. & Chai, J. A review of characteristics of landfilled municipal solid waste in several countries: Physical composition, unit weight, and permeability coefficient. *Polish J. Environ. Stud.*, **27**(6) (2008).

[41] Yang R, Xu Z, Chai J (2020). Numerical analysis of three-dimensional infiltration in a municipal solid waste landfill under rainfall. Pol. J. Environ. Stud..

[42] Coli N, Pranzini G, Alfi A, Boerio V (2008). Evaluation of rock-mass permeability tensor and prediction of tunnel inflows by means of geostructural surveys and finite element seepage analysis. Eng. Geol..

[43] Du X, Zeng YW, Tang DY (2010). Research on permeability coefficient of rock mass based on underwater pumping test and its application. Chin. J. Rock Mech. Eng..

[44] Park D, Oh J (2018). Permeation grouting for remediation of dam cores. Eng. Geol..

[45] Yang FR, Lee CH, Kung WJ, Yeh HF (2009). The impact of tunneling construction on the hydrogeological environment of “Tseng-Wen Reservoir Transbasin Diversion Project” in Taiwan. Eng. Geol..

[46] Foyo A, Sánchez MA, Tomillo C (2005). A proposal for a secondary permeability index obtained from water pressure tests in dam foundations. Eng. Geol..

[47] Tang M, Xu Q, Yang H, Li S, Iqbal J, Fu X, Huang X, Cheng W (2019). Activity law and hydraulics mechanism of landslides with different sliding surface and permeability in the Three Gorges Reservoir Area, China. Eng. Geol..

[48] Uromeihy A, Barzegari G (2007). Evaluation and treatment of seepage problems at Chapar-Abad Dam, Iran. Eng. Geol..

[49] Standart CSN 83 8030 (1995) Waste landfilling - Basic conditions for design and construction. Prague: Czech Standards Institute, Validity: 1.5.1995 – 1.3.1998.

[50] Standart CSN 83 8030 (2018). Waste landfilling - Basic conditions for design and construction of landlills. Prague: Czech Standards Institute, Validity: 1.5.2002 – 1.10.2018.

[51] Standard CSN 73 1001 (1988). Foundation of structures. Foundation soil below shallow foundations, Validity: 10/1988 - 03/2010.

[52] Standard CSN 83 8032 (2002). Waste landfilling - Sealing, Validity: 05/2002 - 07/2018.

[53] Standard CSN 72 1006 (1998). Consolidation tests for soil and loose materials. Validity: 01/1999 - 06/2015.

[54] Standard CSN EN ISO 14688–2 (72 1003), 2005. Geotechnical investigations and testing – Identification and classification of soils—Part 2: Rules for classification, Validity: 04/2005 - 04/2018.

